# The kynurenine pathway implicated in patient delirium: possible indications for indoleamine 2,3 dioxygenase inhibitors

**DOI:** 10.1172/JCI164577

**Published:** 2023-01-17

**Authors:** Amy B. Heimberger, Rimas V. Lukas

**Affiliations:** Feinberg School of Medicine, Northwestern University, Chicago, Illinois, USA.

## Abstract

Tryptophan (Trp) metabolism plays a central role in sleep, mood, and immune system regulation. The kynurenine pathway (KP), which is regulated by the enzymes tryptophan 2,3-dioxygenase (TDO) and indoleamine 2,3 dioxygenase (IDO), which catalyze the conversion of Trp to kynurenine (Kyn), facilitates immune regulation and influences neurocognition. Notably, Kyn metabolites bind the *N*-methyl-d-aspartate receptor (NMDAR), essential for memory encoding, and in turn, cognition. Aberrant NMDAR activity through agonist binding influences excitability and cell death. In this issue of the *JCI*, Watne and authors demonstrate that KP pathway end products were elevated in the serum and the cerebrospinal fluid (CSF) of subjects with delirium. This observation provides insight regarding the basis of a variety of commonly observed clinical conditions including sundowning, abnormal sleep-wake cycles in hospitalized patients, neurodegenerative cognitive impairment, radiation-induced cognitive impairment, neurocognitive symptomatology related to COVID-19, and clinical outcomes observed in patients with CNS tumors, such as gliomas.

## Tryptophan metabolism and the kynurenine pathway

**Figure 1:**
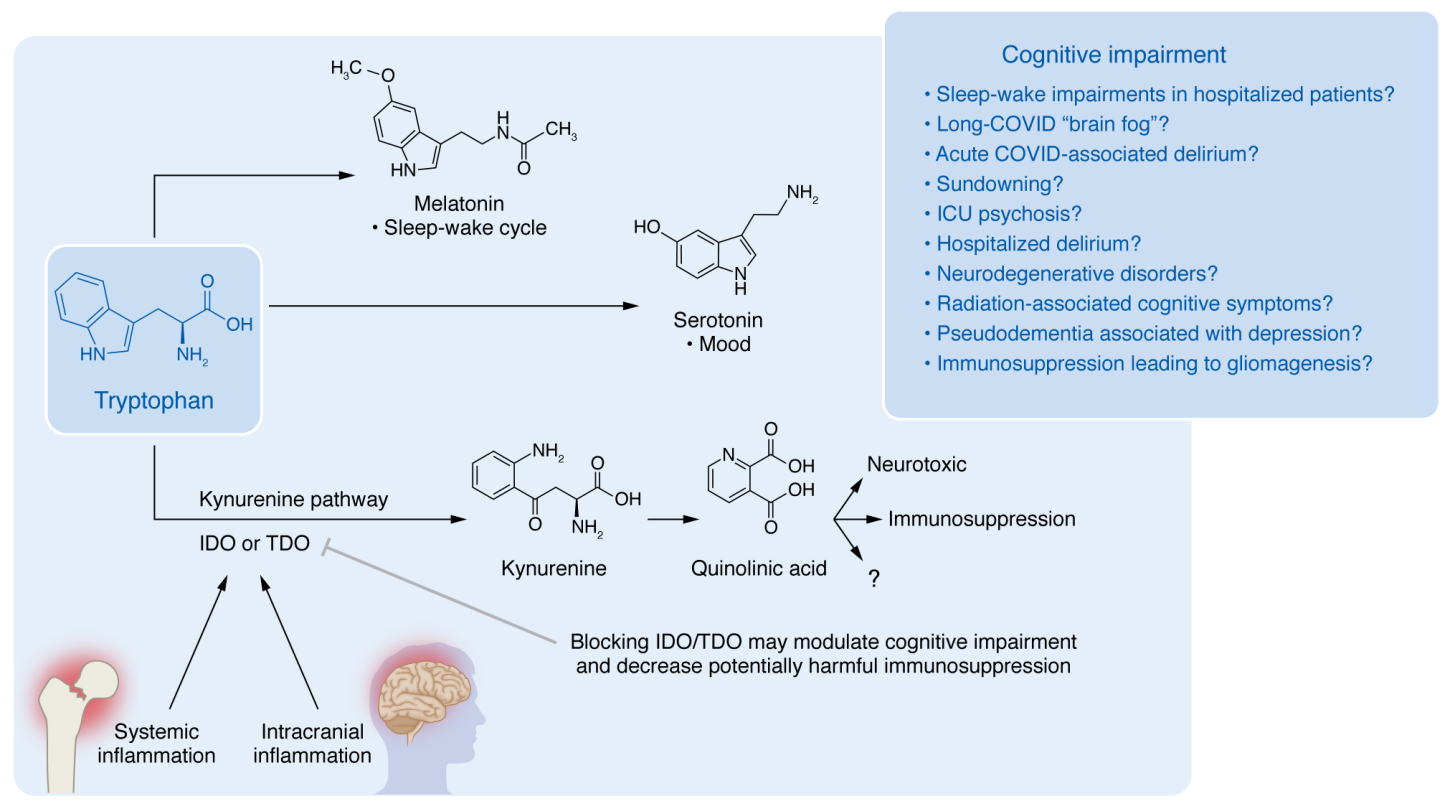
Trp metabolism and the KP associate with delirium. Trp can be metabolized into melatonin, serotonin, and Kyn. While the KP plays a role in suppressing the immune system, the QA metabolite also shows evidence of direct neurotoxicity. Both Trp metabolism and KP mechanisms may account for the cognitive impairment that associates with various conditions, including hospitalized delirium (4). Modulating the KP via IDO or TDO inhibition may mitigate negative neurocognitive outcomes associated with a variety of conditions.

Tryptophan (Trp) is an essential amino acid that can be metabolized into melatonin, which facilitates the sleep-wake cycle, and serotonin, which is essential for regulating mood. In addition, Trp has long been known to play a role in immune regulation via its metabolism along the immunosuppressive kynurenine (Kyn) pathway (KP). Specifically, the KP leads to the downstream production of immunosuppressive 3-hydroxykynurenine (3-HK), 3-hydroxyanthranilic acid (3-HAA), and quinolinic acid (QA) (Figure 1).

KP metabolites may also agonize or antagonize the *N*-methyl-d-aspartate receptor (NMDAR), which functions in memory encoding and cognition. Agonist binding can lead to excitotoxicity triggering neuronal death or receptor downregulation and diminishing signal transduction (1, 2). These interactions also compromise blood-brain barrier integrity (3). However, a full understanding of the factors facilitating specific effects remains elusive.

In this issue of the *JCI*, Watne et al. report the results of a multiinstitutional study of paired serum and cerebrospinal fluid (CSF) samples from 586 hospitalized patients with or without delirium. Subjects experiencing delirium had higher concentrations of QA and other products of KP activity (4). The authors proposed that systemic inflammation activates indoleamine 2,3 dioxygenase (IDO), which can be induced by interferon signaling and is expressed in a variety of cells, including those that reside in the brain. KP activity is also stimulated by tryptophan 2,3-dioxygenase (TDO), which is primarily expressed in the liver and is induced by glucocorticoids and glucagon. IDO and TDO enzyme activities promote the conversion of Trp down the immunoregulatory KP, theoretically leaving less available for metabolism in the melatonin and serotonin pathways, which are integral to sleep and mood, respectively. IDO and TDO enzyme activities increase Kyn levels, which can be measured in the CSF, a proxy indicator of intraparenchymal brain concentrations. Results showing a correlation of serum and CSF levels with the presence of delirium introduce the possibility of a readily accessible serum delirium biomarker (4).

Having only one analyzed time point limits interpretation of the Watne et al. study (4), thereby compromising assumptions about relationships between the kinetics of KP activity and delirium. Addressing this concern would require longitudinal assessments through multiple serum blood and CSF draws or the use of CNS-implanted microdialysis catheters or lumbar drains. However, it should be considered that these techniques may only roughly approximate concentrations in brain parenchyma. An additional limiting factor for interpreting the study results is that the findings were correlative and did not demonstrate causality. However, preclinical data have previously shown that IDO inhibitors and IDO deficiency in mice protect against cognitive dysfunction (5, 6). Previous work has additionally shown that the injection of QA in the brains of animals induces hyperactive behavior and impairs memory (7). Watne et al. (4) also report that the levels of CSF QA correlated with the neuronal damage marker neurofilament light chain. This result suggests the potential for longer term sequelae of episodic delirium (Figure 1).

Because IDO inhibitors are being extensively investigated in cancer patients, clinical trials that include delirium status assessments in the study design would likely address an important clinical need of determining an impact on cognitive function. The results from Watne et al. (4) also suggest that there is therapeutic potential for the use of IDO inhibition in various circumstances, including in the delirious patient population, for mitigation of sleep-wake cycle disturbances, for delaying neurodegenerative disorder progression, and for lessening neurologic damage and cognitive impairments from radiation therapy.

## KP products are associated with disease

The upregulation of Trp and subsequent increase in downstream KP products are associated with aging (8) and an increased risk of age-dependent neurodegenerative disorders, such as Parkinson’s disease (9). This correlation may be a consequence, in part, of increased expression of IDO that occurs with increasing age in the healthy brain (10). Increased IDO could contribute to the frequently observed phenomena of delirium and sundowning in elderly hospitalized patients. These observations support the potential benefit of studies investigating IDO inhibitors for treating various age-related neurodegenerative conditions.

KP activity in the context of CNS cancer, especially glioblastoma (GBM), suppresses cytotoxic T cells to inhibit immune anticancer function. The use of IDO inhibitors has been and continues to be investigated for treating GBM to offset the protumor effects of elevated KP activity and for potential synergistic antitumor activity when combined with radiation therapy and/or immune checkpoint inhibitors (11). Preclinical studies have shown that pharmacologic inhibition of IDO synergizes with chemo-radiation therapy to trigger long-term survival in mice bearing intracranial GBM. Mice genetically deficient in complement component C3 do not benefit from IDO inhibitor treatment, suggesting C3 as an important mediator of IDO inhibitor effects (12).

An epidemiologic analysis of the National Cancer Institute Surveillance, Epidemiology, and End Results (SEER) (https://seer.cancer.gov/), the Broad Institute’s Genotype Tissue Expression project (https://gtexportal.org/home/), and the University of California San Francisco’s 10k Immunomes (https://comphealth.ucsf.edu/app/10kimmunomes) databases for associations between gene expression and aging reveals that the expression of factors associated with immunoregulation, including IDO1 and the immune checkpoint therapeutic target programmed death-ligand 1, increase in normal brain as it ages, and these increases are coincident with increased circulating immunosuppressive Tregs as well as decreased cytolytic CD8^+^ T cells in peripheral blood. These alterations were most pronounced in individuals in their seventh decade, coincident with the peak incidence for the development of GBM (10). These associations provide some rationale for the lack of efficacy of some immunotherapeutic approaches for treating GBM and are consistent with results from preclinical studies showing reduced effectiveness of immunotherapy in treating advanced age in mice with intracranial tumor when compared against corresponding results from younger mice (13). Such preclinical data suggest that clinical trials using IDO inhibitors in advanced age GBM may yield mostly negative results or may require additional therapeutic combinations to overcome age-related alterations. Decreased immune surveillance and an overall immunosuppressed microenvironment in the CNS in association with increasing age may also provide some explanation for the increasing incidence of CNS cancer with increasing age.

Radiotherapy, a longstanding cornerstone of the treatment of cancer, is well documented for its negative developmental effects when used in treating children (14). Negative cognitive and quality-of-life effects from radiotherapy have also been extensively documented in elderly patients (15). Multiple radiation strategies have been devised to overcome radiotherapy side effects that include the use of more focal radiation and the use of NMDAR antagonists such as memantine (16, 17). In the phase III NRG CC001 trial (*n* = 581), adult patients with brain metastases were randomized to either traditional whole-brain radiotherapy (WBRT) plus memantine or hippocampal-avoidance WBRT (HA-WBRT) plus memantine. The primary end point of the trial was time to cognitive function failure, defined as decline. While overall survival, intracranial progression-free survival, and toxicity were equivalent in the two arms, cognitive decline was significantly reduced by the combination of memantine with HA-WBRT (adjusted hazard ratio, 0.74; 95% CI, 0.58 to 0.95; *P* = 0.02). This difference included reduced deterioration in executive function at 4 months (23.3% versus 40.4%; *P* = 0.01) as well as learning and memory at 6 months (11.5% versus 24.7% [*P* = 0.049] and 16.4% versus 33.3% [*P* = 0.02], respectively). At 6 months, patients receiving HA-WBRT plus memantine reported statistically significant reductions in fatigue, speech impairment, and interference with daily activities (*P* = 0.01) (17). The benefit observed in the NRG CC001 trial may be due to a combination of decreased neuronal excitotoxicity by NMDAR antagonism with decreased direct neuronal injury and/or local inflammation by sculpting the radiation field to avoid critical structures. These successes support the value of pursuing additional approaches for decreasing the damaging effects of radiotherapy on neurocognitive function, including upstream modulation of KP activity through use of an IDO inhibitor.

## Conclusion and future directions

The Watne et al. report (4) provides support for a direct relationship between KP activity and delirium. Particularly exciting are the potential applications of IDO inhibitors in lessening the negative neurocognitive effects of a number of common clinical scenarios ranging from delirium in the hospitalized patient to radiotherapy for CNS tumors to COVID-related cognitive impairment. Related clinical studies could move forward quickly, with extensive safety and toxicity support already available through oncology research. Several neurodegenerative diseases could also potentially benefit from KP activity suppression through use of IDO and or TDO inhibitors. Increasing clinical research with IDO and TDO inhibitors could as well prove important in the development of treatment strategies for activating immune function against CNS cancer. Study design inclusion of secondary end points involving neurocognitive function would be essential for addressing the potential benefits of PK activity modulation in treating a variety of clinical conditions.

## Version 1 01/17/2023

Electronic publication
